# Insufficient utilization of care in male incontinence surgery: health care reality in Germany from 2006 to 2020 and a systematic review of the international literature

**DOI:** 10.1007/s00345-023-04433-9

**Published:** 2023-06-01

**Authors:** Martin Baunacke, Elena Abbate, Nicole Eisenmenger, Ulrich Witzsch, Angelika Borkowetz, Johannes Huber, Christian Thomas, Juliane Putz

**Affiliations:** 1 Department of Urology, Medical Faculty Carl Gustav Carus, TU Dresden, Fetscherstr. 74, 01307 Dresden, Germany; 2 Reimbursement Institute, Hürth, Germany; 3 Department of Urology, Krankenhaus Nordwest, Frankfurt, Germany; 4 Department of Urology, Philipps-University Marburg, Marburg, Germany

**Keywords:** Incontinence surgery, Postprostatectomy incontinence, Artificial sphincter, Slings, Health care

## Abstract

**Purpose:**

Data suggest that the utilization of care in male incontinence surgery (MIS) is insufficient. The aim of this study was to analyse the utilization of care in MIS from 2006 to 2020 in Germany, relate this use to the number of radical prostatectomies (RP) and provide a systematic review of the international literature.

**Methods:**

We analysed OPS codes using nationwide German billing data and hospitals’ quality reports from 2006 to 2020. A systematic review was performed according to the Preferred Reporting Items for Systematic Review and Meta-Analyses (PRISMA).

**Results:**

MIS increased by + 68% from 2006 to 2011 (1843–3125; *p* = 0.009) but decreased by − 42% from 2011 to 2019 (3104–1799; *p* < 0.001). In 2020, only 1435 MISs were performed. In contrast, RP increased from 2014 to 2019 by 33% (20,760–27,509; *p* < 0.001). From 2012 to 2019, the number of artificial urinary sphincters (AUSs) changed minimally (− 12%; 1291–1136; *p* = 0.02). Sling/sling systems showed a decrease from 2011 to 2019 (− 68% 1632–523; *p* < 0.001). In 2019, 63% of patients received an AUS, 29% sling/sling systems, 6% paraurethral injections, and 2% other interventions. In 2019, few high-volume clinics [*n* = 27 (13%)] performed 55% of all AUS implantations, and few high-volume clinics [*n* = 10 (8%)] implanted 49% of retropubic slings.

**Conclusion:**

MIS have exhibited a relevant decrease since 2011 despite the increase in RP numbers in Germany, indicating the insufficient utilization of care in MIS. The systematic review shows also an international deficit in the utilization of care in MIS.

**Supplementary Information:**

The online version contains supplementary material available at 10.1007/s00345-023-04433-9.

## Introduction

Male stress urinary incontinence (SUI) is predominantly iatrogenic. The most common reason is radical prostatectomy (RP) [1]. Incontinence rates after RP vary between 4 and 31% depending on follow-up time and incontinence definition [2]. Healthcare research data show an incontinence rate of 15% 6 years after RP in Germany [3].

Urinary incontinence significantly impairs quality of life [4]. After unsuccessful conservative treatment, several surgical procedures are available to treat male urinary incontinence. Artificial urinary sphincters (AUS) and slings are predominantly used, depending on the severity of incontinence [5, 6]. These surgical implants show good results in improving incontinence symptoms and quality of life [7]. To date, the artificial sphincter is the gold standard in the therapy of moderate to severe SUI [8]. Slings and sling systems are recommended for men with mild to moderate SUI [8]. Other less commonly used treatments are paraurethral bulking and an adjustable balloon system (PROAct^®^) [9]. The indication for surgery is primarily a reduced quality of life because of SUI. Although not every incontinent man needs surgery, data suggest that utilization of care is insufficient in male incontinence surgery (MIS) [5, 10-13]. Specifically, register data show a low proportion of incontinence surgery in cohorts of patients after RP in relation to incontinence rates [6, 11-14]. Although few studies exist on this topic, they show comparable results in different countries.

The aim of this study was to analyse the utilization of care in MIS from 2006 to 2020 in Germany and to provide a review of the international literature concerning insufficient utilization of care in MIS after RP.

## Materials and methods

We used two datasets to analyse the development of MIS. To represent the longitudinal course, we used Diagnosis Related Groups (DRG) billing data from DESTATIS (Statistisches Bundesamt), which include information about age and sex. For the cross-sectional view in 2019, we used quality reports of German hospitals. They have been mandatory since 2006 and are published annually since 2012. To analyse procedures, we used OPS (Operationen- und Prozedurenschlüssel) codes. Specifically, we used the following codes: AUS: 5–597.0, 5–597.30, 5–597.31, 5–597.32; sling or sling systems: 5–594, 5–596.70, 5–598.0/x; adjustable continence therapy: 5–596.74 (bladder neck), 5–596.75 (bulbar urethra); paraurethral injection: 5–596.0, 5–596.00, 5–596.01, 5–596.02, 5–596.0x; others: 5–596.x/y, 5–598.x/y. For RP, we used the following code: 5–604.

We used quality reports to analyse the structural health care situation in Germany in 2019 and 2012. Here, 1–3 cases per year were anonymized as a case number of 1, differing slightly from the DESTATIS data. Furthermore, gender could not be differentiated. DESTATIS data allow male and female incontinence surgery to be distinguished. Therefore, we analysed three codes as representations for structural development in 2019. We used the code for AUS. The rate of female usage was 1.6% and consequently negligible. We also used 5–598.0/x for male slings. We included Code 5–596.75 for sling analysis in 2019. Again, the rate of female usage was only 0.7% and also negligible. We did not use Code 5–594 because the proportion of male patients was low (2.3%). The clinics were divided into high-volume (≥ 10 cases/year) and low-volume (< 10 cases/year) groups.

We used the analysis tool “reimbursement.INFO” (RI Innovation GmbH, Hürth, Germany) to extract these data from quality reports and DESTATIS on male incontinence surgery for the years 2006–2020. Because of the coronavirus pandemic and consequent reduction in elective surgery in Germany, we focused on development between 2006 and 2019, reflecting an undisturbed health care reality [15].

A systematic review was performed according to the Preferred Reporting Items for Systematic Review and Meta-Analyses (PRISMA). The objective was to search for the rate of MIS after RP in the international literature. One reviewer searched PubMed.gov two times for the combined terms “incontinence surgery” and “radical prostatectomy” until December 2, 2022. References were screened by title and abstract. All studies that showed the rate of MIS after RP were included. Studies differed by database studies and patient surveys. Patient survey studies were analysed regarding incontinence-related quality of life and usage of MIS.

Due to the nature of the data (publicly accessible hospital quality reports and data from DESTATIS), ethical approval was not needed. We applied t tests and linear regression analyses to analyse the trends over time. *p* < 0.05 was considered to indicate significance. All calculations were performed with “IBM SPSS Statistics 28” (Armonk, NY, USA).

## Results

### Development over time

**Fig. 1:**
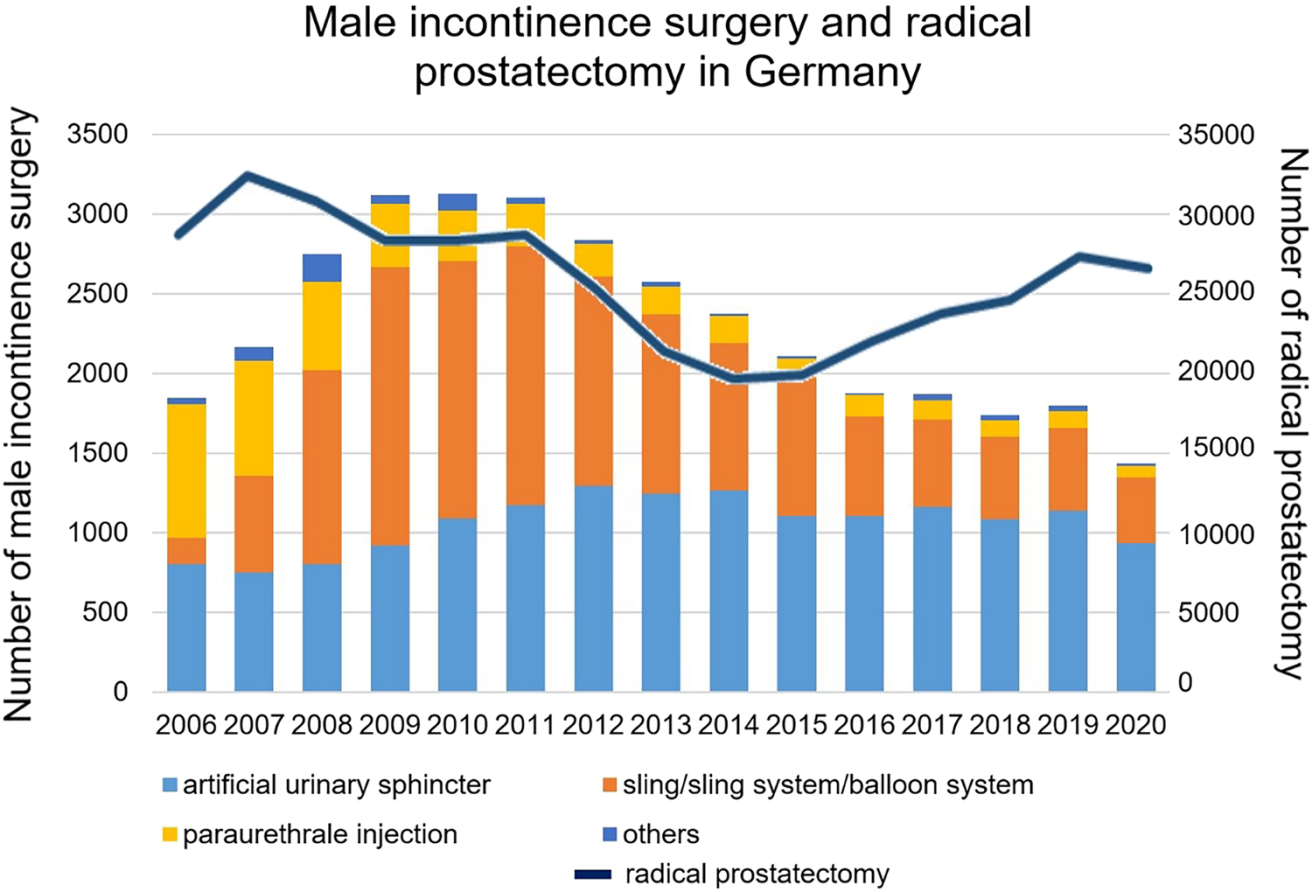
Numbers of incontinence surgeries and radical prostatectomy from 2006 to 2020 in Germany

The rate of MIS increased by + 68% from 2006 to 2011 (from 1843 to 3125; + 273 ± 58 interventions/a; *p* = 0.009) but decreased by − 42% from 2011 to 2019 (from 3104 to 1799; − 174 ± 19 interventions/a; *p* < 0.001). In 2020, only 1435 MIS were performed due to the coronavirus pandemic (Fig. 1). Regarding the age distribution of patients, the proportion of older patients increased from 2006 to 2019: ≥ 80 years: 5–15% (*p* < 0.001), 70–79 years: 45–54% (*p* < 0.001). The proportion of patients aged 60–69 years decreased from 40–24% (*p* < 0.001). The proportion of young patients (< 50 years) was very low and remained unchanged (3% in 2006, 2% in 2019; *p* = 0.09) (Suppl. Fig. 1).

### Incontinence surgery

In 2019, 1799 MIS were performed. A total of 63% of patients received an AUS, 29% received a sling/sling system, 6% received paraurethral injections, and 2% received other injections (Fig. 1). Different developments in surgical procedures have emerged over time. The prevalence of AUS increased from 2006 to 2012 (+ 61%; 804–1291; *p* < 0.001). From 2012 to 2019, the numbers of AUS only changed minimally (− 12%; 1291–1136; *p* = 0.02). A detailed analysis of AUS shows a decreasing proportion of two-cuff systems in comparison with one-cuff systems from 2006 to 2019 (32% vs. 14%; *p* < 0.001). The sling/sling system showed a strong increase from 2006 to 2011 (+ 896%; 163–1623; *p* < 0.001) and a strong decrease from 2011 to 2019 (− 68% 1632–523; *p* < 0.001). Analysing sling/sing system codes showed the highest number of suprapubic slings in 2008, with a significant decrease to 2019 (674–24; *p* < 0.001), and adjustable systems also decreased from their highest number in 2009 to 2019 (921149; *p* < 0.001). In 2019, the localization could be differentiated. Nineteen adjustable procedures were performed on the bladder neck (PROAct^®^) and 130 on the bulbar urethra in 2019 (Suppl. Figure 2). Paraurethral injections decreased continuously from 2006 to 2019 (− 88%; 838–103; *p* < 0.001). In 2019, the proportions of substances were as follows: 42% dextranomer/hyaluronic acid, 28% polyacrylamide hydrogel, 15% silicone, and 15% others.

### Comparison with radical prostatectomy numbers

In 2007, the prevalence of RP peaked with 31,945 procedures and decreased thereafter from 2007 to 2014 (− 35%; 31,945–20,760; *p* < 0.001). This decrease corresponds with a decrease in incontinence surgery. From 2009 to 2014, the proportions of MIS and RP decreased by − 24% and − 27%, respectively. However, from 2014 to 2019, the proportion of RP increased by + 33% (20,760–27,509; *p* < 0.001), which was contrary to the trend in incontinence surgery, with a decrease of − 24% (2376–1799; *p* < 0.001) (Fig. 1). The development of age distribution in RP corresponds to that of age distribution in incontinence surgery. The proportion of older patients increased from 2006 to 2019: ≥ 80 years: 0.1–1% (*p* < 0.001), 70–79 years: 23–33% (*p* < 0.001). The proportion of patients aged 60–69 years decreased from 59 to 48% (*p* < 0.001). The proportion of young patients (< 50 years) was very low and changed minimally (1.1% in 2006, 1.6% in 2019; *p* < 0.001).

### Structural health care situation in Germany

Suppl. Table 1 shows the structural health care situation in Germany using the example of AUS and retropubic slings. No difference in AUS was detected between 2019 and 2012, showing that few high-volume clinics [*n* = 27 (13%)] performed 55% (*n* = 574) of all AUS implantations in 2019. Regarding slings, a decrease in high-volume clinics was observed from 2012 to 2019. In 2019, few high-volume clinics [*n* = 10 (8%)] implanted 49% of retropubic slings in comparison with 2012, when more high-volume clinics [*n* = 29 (16%)] implanted 61% of all slings.

### Systematic review of MIS rate after RP in international literature

The search retrieved 188 references. Eleven studies reported the rate of MIS after RP (Table 1). Two studies used the same cohort with different subsequent analyses [6, 16]. Nine studies were database analyses showing the share of incontinence surgery in a large cohort of patients after RP from the USA, Canada, Austria and Sweden [6, 11-14, 16-19]. Two studies of patient surveys provided more detail [5, 10]. The use of incontinence surgery after RP ranged between 2.5 and 3.9%. One study showed a higher rate of incontinence surgery of 6% in a selected cohort of older patients (age at RP > 65 years) [12].

**Table 1:** Review of international literature regarding the rate of incontinence surgery after RP

First author [Reference]	Year	Country	Year of RP	Number of RP	Rate of incontinence surgery	Comments
Wehrberger [13]	2012	Austria	1992–2009	33,580	2.8%	Database study
Nam and Wallis [6, 16]	2012	Canada	1993–2006	25,346	3.9%	Database study
Kim [12]	2013	USA	2000–2007	16,348	6%	Database study Collective > 65 years
Gupta [18]	2016	North Carolina/USA	1998–2012	4401	3.7%	Database study
Ventimiglia [14]	2017	Sweden	2000–2014	26,280	3%	Database study
Nelson [11]	2020	Florida/USA	2006–2015	29,287	3.6%	Database study
Baunacke [5]	2020	Germany	2008–2013	936	2.7%	Patient survey of 936 men 14% ≥ pads/day 25% of all SUI patients underwent surgery 46% of SUI patients with a need to treat underwent surgery
Kim [17]	2021	Florida/USA	2008–2010	13,535	3.3%	Database study
Parry [10]	2022	UK	2014–2016	11,290	2.5%	Database study of 11,290 men and patient survey of 5,165 men 9.3% poor EPIC-26 urinary incontinence score 9.1% of all SUI patients underwent surgery 20% of SUI patients with a need to treat underwent surgery
Del Giudice [19]	2022	USA	2003–2017	21,589	3.4%	Database study

## Discussion

The prevalence of MIS increased by + 68% from 2006 to 2011 in Germany but decreased by − 42% from 2011 to 2019 due to a significant decline in the number of sling implantations. In contrast to the decline in the number of MIS, the number of RPs increased from 2014 to 2019.

This study is the first to show the development and health care reality of MIS in detail in a Western country. In 2019, the predominant share of MIS in Germany was AUS (63%). Sling/sling systems had a share of 29%. Few studies have examined the distribution of surgical procedures. In a British study, 67.9% of patients received AUS, and 30.3% received slings [10]. In a German study of a small population (*n* = 26) that underwent RP between 2008 and 2013, 38% received an AUS, and 68% received slings/sling systems [5]. In the years between 2009 and 2011, a similar distribution was identified in our study, with 30–38% AUS and 52–56% slings. In Germany, the structural health care reality in 2019 shows that approximately 50% of all AUS or slings are implanted in high-volume clinics (Suppl. Table 1), which is congruent with earlier data [20]. This finding shows a kind of centralization. Nevertheless low-volume clinics (< 10 cases/year) implant the remaining 50% of all AUS or slings. Due to the relevant complication and revision rate, centralization with correspondingly higher surgical experience is advocated [21].

The longitudinal course of MIS in Germany has shown a decline of − 42% since 2011, resulting mainly from a decrease in sling surgery. A US database analysis of MIS between 2003 and 2013 also shows a peak of sling surgery in 2011 (62.2% slings vs. 37.8% AUS) with a subsequent decline [22]. Whether the US Food and Drug Administration (FDA) mesh ban in 2013 had an impact on this rate remains unclear [23]. The differences in the distribution of both procedures between these studies may result from the different financial resources of these countries’ health care systems. This peak of male sling surgery may result from the introduction of new sling and sling systems. Subsequent studies showed limitations of slings and led to current recommendations for mild to moderate SUI without previous radiotherapy, which may explain the decline in sling surgery [7, 8]. The introduction of male slings replaced paraurethral bulking.

Even if a “sling hype” existed between 2009 and 2011 with a subsequent optimization of indication resulting in a decrease in sling numbers, a major question is to be answered: Where have the patients with SUI gone? Specifically, − 42% fewer MIS were performed in 2019 than in 2011. Inferior results of slings may led to the decrease in sling surgery [24]. However, a compensatory increase in AUS as the alternative treatment was not observed. So there seemed to be that MIS is underutilized.

Although RP is the most common cause of male SUI, it is not the only cause [1]. Therefore, we compared these data with the numbers of RPs. Assuming an incontinence rate of 15% after RP, there seemed to be a gap in the number of MIS comparing the number of RP [3]. Overlaying both graphs show a synchronous decrease until 2014 (Fig. 1). After 2014, the developments are the opposite. Several studies showed a median time between RP and incontinence surgery of 3 years [10, 14, 25]. Considering this timespan, a continuous increase in MIS should have occurred since 2017. To explain the gap between the numbers of RPs and MISs, we assume that the utilization of care in male incontinence surgery is insufficient.

Our review of the literature shows that international studies also highlight the problem of insufficient utilization of care in MIS. Studies discuss an insufficient utilization of care in male incontinence surgery considering a higher incontinence rate resulting in a gap in nontreated patients. The use of incontinence surgery after RP ranges between 2.5 and 3.9%. One study with a rate of 6% was performed in a selected cohort of older patients (age at RP > 65 years) biased by higher age, resulting in a higher incontinence rate after RP [12, 26]. The suspected insufficient utilization in database studies is confirmed by two patient survey studies [5, 10]. Both studies show an incontinence rate after RP of 2.5–2.7%. A British study showed that 9.1% of patients with a low EPIC-26 urinary incontinence score underwent incontinence surgery [10]. A German study showed that 25% of patients with ≥ 2 pads/day underwent incontinence surgery (rate of incontinence surgery after RP: 2.7%) [5]. Quality of life and personal suffering from incontinence are relevant for surgical treatment. The British study shows that only 20% of patients with a need for surgery are reached [10]. A German publication reports only 46% of patients who show poor parameters in incontinence-related quality of life underwent with MIS [5]. Both studies underline the problem of insufficient utilization of care in male incontinence surgery. All eleven studies reveal an insufficient utilization that seemed not to depend on very different health care systems in all six western countries [5, 6, 10-14, 16-19].

This database study is subject to limitations. Our study data are based on OPS coding, which is partly not very specific. Coding depends on the surgeon and clinic. In particular, coding for slings varies. Furthermore, in 2009, ambiguities existed in using AUS code for adjustable slings. This practice was curbed by health insurance companies in the following years. Nevertheless, DRG data enable a very accurate representation of the health care situation. Hospital quality reports are less exact because of the anonymization of case numbers ≤ 3. Another limitation is the lack of clinical data. RP is the most common cause of male SUI, but the number of incontinence surgeries caused by RP cannot be shown. Moreover, the share of patients with multiple MIS also cannot be shown.

Nevertheless, this study shows MIS in a large Western country in detail and reflects its usage over 14 years. Specifically, this study shows the development of a relevant gap in the utilization of MIS in the last 10 years that cannot be explained by the development of RP numbers. A German study showing insufficient treatment of stress urinary incontinence after RP showed no difference of incontinent patients with or without MIS in age and Charlson score. Therefore, no medical reasons, such as high age or severe comorbidity, explain the lack of surgical treatment [5]. Studies have demonstrated deficits in communication between patients and physicians concerning male incontinence. A study shows that the extent of urine leakage is often underestimated by doctors [27]. A large US study showed that the impairment of the quality of life of incontinent men was significantly underestimated by physicians [28]. On the other hand, a study showed that 75% of incontinent men wanted to discuss their problem with their doctor. However, in the end only 32% did it [29]. Nevertheless, further studies are needed to precisely determine the reasons for this deficit to develop interventions to improve the level of care.

## Conclusion

A relevant decrease in MIS has been observed since 2011, despite an increase in RP numbers. This decrease could be an indication of the insufficient utilization of care in MIS. A review of the literature also shows an international deficit in the utilization of care in MIS with consistent results. We need to investigate the reasons for this insufficient utilization of care to develop interventions to improve the level of care for MIS.

## Supplementary Information

Below is the link to the electronic supplementary material.

Suppl. Figure 1: Age distribution of male incontinence surgery from 2006 to 2020 in Germany

Suppl. Figure 2: Distribution of OPS codes regarding slings and sling systems from 2006 to 2020 in Germany

Supplementary file3 (DOCX 13 KB)

## Data Availability

Data publicly available in a repository: Hospital quality reports are available at the following URL: https://www.g-ba.de/themen/qualitaetssicherung/datenerhebung-zur-qualitaetssicherung/datenerhebung-qualitaetsbericht/.
