# Supplementary figures and images for: Insufficient utilization of care in male incontinence surgery: health care reality in Germany from 2006 to 2020 and a systematic review of the international literature
Source: World J Urol. 2023 Jun 1;41(7):1813–9. doi: 10.1007/s00345-023-04433-9 (PMC10233526; doi:10.1007/s00345-023-04433-9)

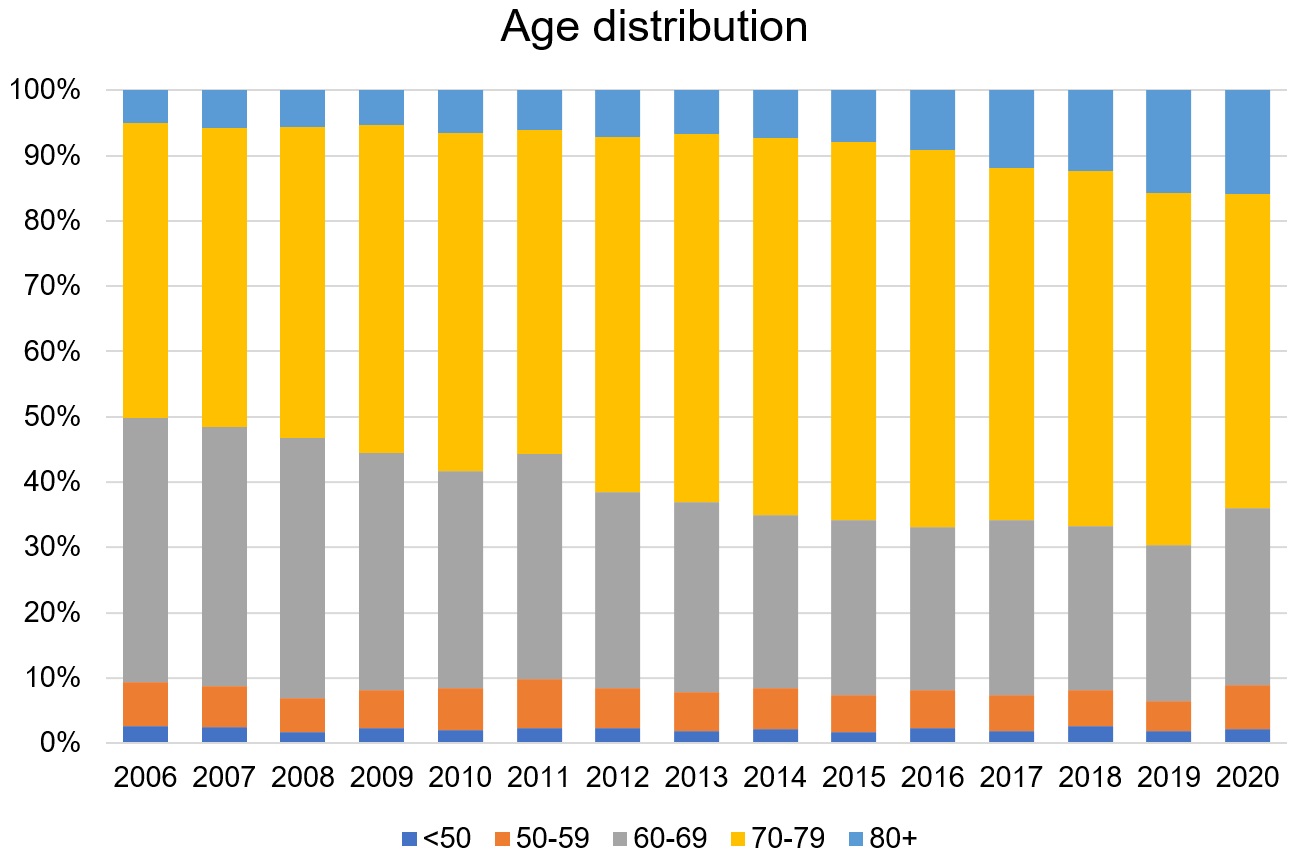

Supplement: Supplementary file 1 — Suppl. Figure 1: Age distribution of male incontinence surgery from 2006 to 2020 in Germany [file 345_2023_4433_MOESM1_ESM.jpg]

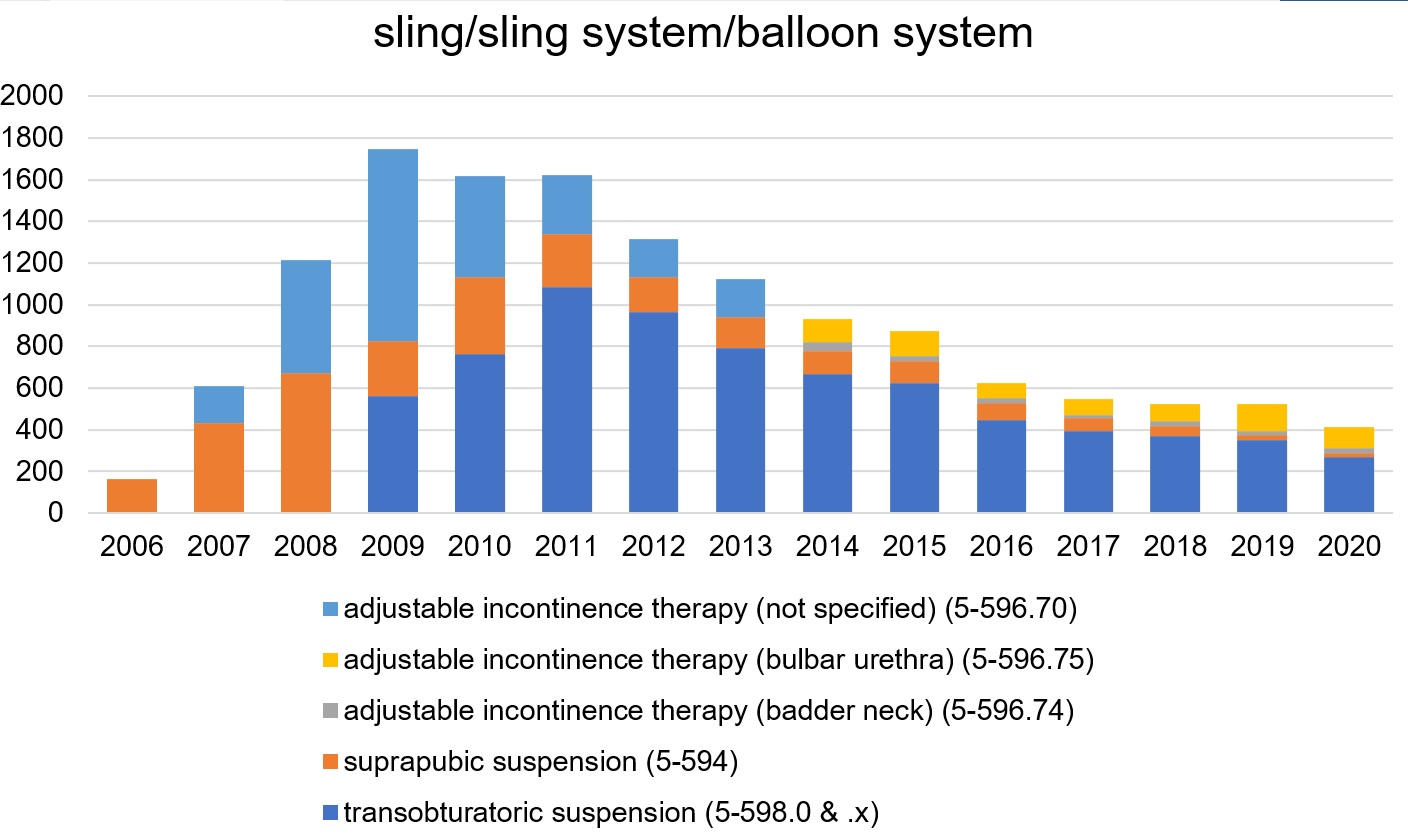

Supplement: Supplementary file 2 — Suppl. Figure 2: Distribution of OPS codes regarding slings and sling systems from 2006 to 2020 in Germany [file 345_2023_4433_MOESM2_ESM.jpg]
