# Supplementary material for: Insufficient utilization of care in male incontinence surgery: health care reality in Germany from 2006 to 2020 and a systematic review of the international literature
Source: World J Urol. 2023 Jun 1;41(7):1813–9. doi: 10.1007/s00345-023-04433-9 (PMC10233526; doi:10.1007/s00345-023-04433-9)
Supplement: Supplementary file 3 — Supplementary file3 (DOCX 13 KB) [file 345_2023_4433_MOESM3_ESM.docx]

| Artificial urinary sphincter | | | | |  | Sling/sling system | | | | |
| --- | --- | --- | --- | --- | --- | --- | --- | --- | --- | --- |
| Year |  | Total | High volume | Low volume |  | Year |  | Total | High volume | Low volume |
| 2012 | Clinics | 209 | 29 (14%) | 180 (86%) |  | 2012 | Clinics | 185 | 29 (16%) | 156 (84%) |
|  | Procedures | 1101 | 602 (55%) | 499 (45%) |  |  | Procedures | 892 | 544 (61%) | 348 (39%) |
| 2019 | Clinics | 202 | 27 (13%) | 175 (87%) |  | 2019 | Clinics | 126 | 10 (8%) | 110 (92%) |
|  | Procedures | 1035 | 574 (55%) | 461 (45%) |  |  | Procedures | 412 | 200 (49%) | 212 (51%) |

**Suppl. Table 1.** Structural health care situation (number of clinics and procedures) in Germany in 2012 and 2019.
